# Binge Drinking in Spanish University Students: Associated Factors and Repercussions: A Preliminary Study

**DOI:** 10.3390/ijerph16234822

**Published:** 2019-11-30

**Authors:** Manuel Herrero-Montes, Cristina Alonso-Blanco, María Paz-Zulueta, Carmen Sarabia-Cobo, Laura Ruiz-Azcona, Paula Parás-Bravo

**Affiliations:** 1 International Doctoral School, Universidad Rey Juan Carlos. Alcorcón, 28922 Madrid, Spain; manuelhemo@gmail.com; 2 Department of Physiotherapy, Occupational Therapy, Rehabilitation, and Physical Medicine, University Rey Juan Carlos, Alcorcón, 28922 Madrid, Spain; cristina.alonso@urjc.es; 3 Department of Nursing, Faculty of Nursing, University of Cantabria. Cantabria, 39008, Spain; maria.paz@unican.es (M.P.-Z.); carmen.sarabia@unican.es (C.S.-C.); laura.ruiz@unican.es (L.R.-A.); 4 IDIVAL, Research Health and Bioethics Law Group, GRIDES, 39008 Cantabria, Spain; 5 IDIVAL, Research Nursing Group, 39008 Cantabria, Spain

**Keywords:** binge drinking, alcohol-related disorders, tobacco use, students, alcohol drinking in college, cannabis, psychological test

## Abstract

Alcohol consumption is common among young people. We performed a preliminary cross-sectional study among students (aged 18–30 years) enrolled for the academic year 2018–2019 at the Faculty of Nursing, University of Cantabria (Spain). We collected information on psychological and sociographic factors, tobacco and cannabis uses, and levels of physical activity by AUDIT questionnaires and in person interviews. The aim of our study was to assess the potential of binge drinking (BD) to adversely affect memory and executive function. We recruited 103 students, of whom 85% were female. The alcohol use pattern of slightly more than one-half of the total population was classified as BD. Among BD students, one-fourth were smokers, and nearly one-third had tried cannabis. The mean onset for alcohol use was 15.11 years. Despite our relatively small sample size, our results show that there are strong relationships between BD and both smoking and cannabis use.

## 1. Introduction

Alcohol consumption is common among young people, particularly in the age group of 15 to 30 years of age. At this age, drinking habits are characterized by a high consumption of alcohol over a short period of time, followed by periods of abstinence, which is known as the pattern of the weekend alcoholic, heavy episodic drinking [1], or binge drinking (BD) [2]. In Spain, there is a high prevalence of BD among younger age groups. In men, the group with the maximum prevalence is registered in the people aged between 25 and 29 years (30%) whereas in women, the group with the greatest prevalence are those aged between 20 to 24 years old (20%) [3]. This pattern is not limited to Spanish youth as, according to European data, the level of binge drinking in Spain is within the average of the European Union [4].

Despite the significance of this problem, there is no clear consensus on the definition of BD. In 1995, Wechsler et al., defined BD as the consumption of five or more drinks (four or more for women) on a single occasion over the previous two weeks [5]. Thereafter, in 2004, the National Institute on Alcohol Abuse and Alcoholism (NIAAA) published one of the most internationally accepted definitions of a weekend alcoholic: The consumption of five or more standard units of alcohol (four or more in women) within a two hour interval in the previous two weeks and with periods of abstinence between these episodes [6]. A unit of alcohol is a drink containing 8 to 14 g of pure alcohol, depending on the country where this is measured. In Spain, a standard drink unit (SDU) has 10 g of pure alcohol [7]. Thus, in Spain, the most appropriate definition for BD could be the consumption of six or more alcoholic drinks for men (60 g)—five or more for women (50 g)—on a single occasion (over a two hour period) at least once in the last 30 days, which, besides being similar to the two previous definitions, covers variables of quantity and frequency adapting these to the definition of SDU used in Spain [1]. The negative consequences of this type of alcohol consumption among young people justifies the need for methods to detect the same [8]. The alcohol use disorders identification test (AUDIT) has good psychometric properties to detect problems related with alcohol use among university students [9].

This pattern of consumption is associated with a decline in the central nervous system in animals [10] and with neuropsychological repercussions in human beings [11]. Alcohol affects areas of the brain that are still developing, such as the hippocampus and the prefrontal section [12]. Carbia et al. [13] attributed the vulnerability of the teenage hippocampus with the neurological effects of alcohol, which can result in episodic memory deficits among students who are binge drinkers. Excessive alcohol consumption has been mainly associated with disorders of memory, attention, planning, and executive functions [12,13,14,15,16,17]. Furthermore, morphological disorders have been found regarding the volume of the prefrontal cortex [12,18]. In addition, several studies have found that these repercussions were related with the consumption pattern rather than the amount of alcohol consumed [16,17]. All of the above suggests the importance of deepening our understanding of the associated factors and the repercussions of alcohol consumption, especially among young people, due to the vulnerability of the nervous system towards the toxic effects of alcohol, as well as the possible accumulative effect that any damage can have [15]. 

Furthermore, numerous studies have shown that alcohol consumption patterns in young people are related with demographic characteristics, such as gender [19,20,21], place of residence [4,22], financial situation [21,23,24], living at the family home [20,23,24], academic performance [25], and the consumption of tobacco and other drugs [19,20,26]. Moreover, a significant relationship has been found between young people emotional state, specifically, level of stress [20,27], feelings of depression and anxiety [28], personality traits [29], and their alcohol consumption. In addition to the consumption of tobacco and drugs, BD has also been recognized as contributing towards an unhealthy lifestyle [19,20,26,30,31]. In contrast, sustained exercise plays a role in the modulation of anti-inflammatory effects, as well as in the preservation of the cognitive function in ageing and neuropathological disorders [32], and can also act as a protective factor towards alcohol-induced brain damage. 

The aim of this study was to analyze the relationship between binge drinking among university students and memory and executive function disorders, psychological factors, sociodemographic factors, tobacco and cannabis use, level of physical activity, and AUDIT questionnaire scores.

## 2. Materials and Methods 

### 2.1. Study Design

A cross-sectional study was performed (preliminary study). The study design was approved by the Ethics Committee of Cantabria, Spain (Code: 2015.102). All procedures were conducted according to the Declaration of Helsinki [33] and participants read and signed a written consent form prior to their participation in the study. Data were anonymized and treated confidentially according to the Personal Data Protection Legislation [34].

### 2.2. Participants

This study included all university students enrolled in the 2018–2019 academic year at the Faculty of Nursing of the University of Cantabria (Spain), aged between 18 and 30 years. The exclusion criteria were: (1) A history of severe traumatic brain injuries; (2) neurological and/or psychiatric diseases; (3) dyslexia; (4) color blindness; (5) students with limited Spanish proficiency; and (6) other sensory deficits.

### 2.3. Variables and Measuring Instruments

Data collection included sociodemographic variables, academic variables, physical activity, and information on alcohol, tobacco, and cannabis consumption.

#### 2.3.1. Definition of Binge Drinker

Individuals with a consumption pattern of six or more alcoholic beverages in the case of men (60 g)—five or more for women (50 g)-on a single occasion (over a two-hour period) at least once during the previous 30 days [1]. This criteria was used as it is the most similar to that proposed by the NIAA [6], adapted to the Spanish population. 

#### 2.3.2. Physical Activity

To measure physical activity, the short version of the International physical activity questionnaire (IAPQ) was used. This questionnaire assesses three types of activity: Walking, activities of moderate intensity, and activities of vigorous intensity. The results are expressed in three levels of activity: Low, moderate, and high. The continuous results are presented as MET—minutes/week [35]. One MET is a unit of measure of the rate at which the body expends energy that is based on the energy expenditure while sitting at rest and is equal to 3.5 milliliters of oxygen per kilogram of body weight per minute [36].

#### 2.3.3. Disorders Related to Alcohol Consumption

The alcohol use disorders identification test (AUDIT) was used [37]. This is a self-administered questionnaire with good psychometric properties for the detection of problematic alcohol consumption among university students [8] with the total score ranging between 0 and 40 points. The first eight questions are scored from 0 and 4 and questions 9 and 10 with 0, 2, and 4 points. This is, in turn, divided into three domains: Questions 1 to 3 refer to hazardous alcohol use, questions 4 to 6 refer to symptoms of dependency, and questions 7 to 10 refer to the harmful alcohol use [37]. The three first questions of this test conform the so-called AUDIT-C.

#### 2.3.4. Neuropsychological Tests

A battery of psychological tests was used, and all tests were validated for use in a young population, for the assessment of memory and executive functions. The Logical Memory I Subtest from the Wechsler-III memory scale (WMS-III) was used [38] to examine verbal episodic memory; the Digit Span Test of the Adult Intelligence Scale featured in the Wechsler-III (WAIS-III) scale [39] was used to verify working memory and assess attention and concentration. Furthermore, Parts A and B of the trail making test (TMT) [40] were used to evaluate visuo-motor tracking speed (part A), attention, and mental flexibility (part B). In addition, the Beck-II Depression Inventory (BDI-II) [41] was used to evaluate depressive symptoms as this instrument has demonstrated to have acceptable psychometric properties in Spanish university students [42]. 

### 2.4. Procedure

Recruitment of study participants was performed via informative sessions and posters placed at the university faculty. Those students interested in participating in the study enrolled and were subsequently contacted in order to arrange a meeting. Data collection was performed by two researchers who were previously trained by a psychologist who was an expert in the field, together with a member of the team, to guarantee the correct execution. All the tests were evaluated by both interviewers. A pilot test was performed with the first 10 participants, after which no modifications were made. The data were collected between November 2018 and March 2019. Data collection was performed in two specially prepared rooms in order to ensure a calm environment and privacy for the study participants.

The data collection took approximately 30 min per participant. Participants were classified as either a binge drinker or a non-binge drinker, according to their responses, and following the criteria proposed by Parada et al. [1].

### 2.5. Statistical Analysis

The data analysis incorporated an initial descriptive analysis. For the categorical and discreet variables, the proportions were estimated with their corresponding 95% confidence intervals, according to the Wilson method and using the Pearson’s chi squared test to perform comparisons. Alternatively, the Fisher’s exact test was used when 20% of the fields presented an expected number of cases which was less, or equal to five. For the continuous variables, means were estimated with their standard deviation or medians and interquartile ranges in the case of asymmetric distributions. The Student’s t-test or ANOVA were used to analyze the relation between quantitative variables and categorical variables with two levels; or categorical variables with more than two levels, respectively. The condition of normality was previously confirmed via the Shapiro–Wilk test. All the statistical analyses were performed using the SPSS package v22.0 by IBM (Armonk, NY, USA).

## 3. Results

**Figure 1:**
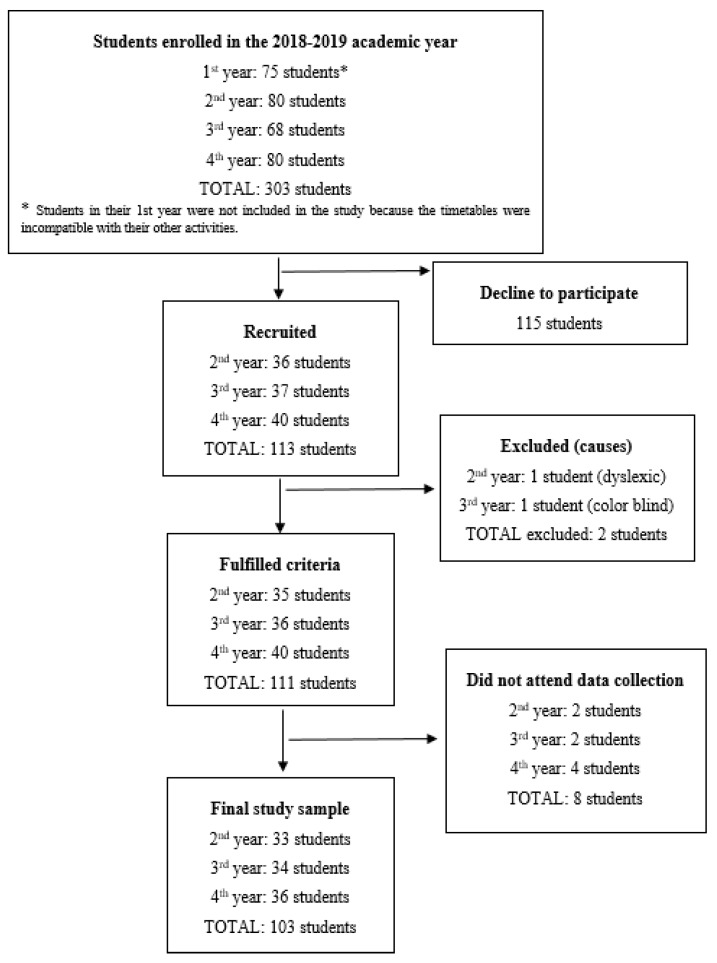
Overall study flow.

In total, 103 participants were included in the study (See Figure 1). According to their pattern of alcohol consumption, 54.37% (*n* = 56) were binge drinkers and 45.63% were non-binge drinkers (*n* = 47). Of the total sample, 84.47% (*n* = 87) were women. The mean age was 21.21 years (SD 2.55). Up to 56.25% of men and 43.68% of women were estimated to be binge drinkers. There was no statistically significant difference between the groups according to gender and age. 

### 3.1. Data on Academic, Sociodemographic, Alcohol Consumption, Tobacco, Cannabis, and Physical Activity Factors

Results are displayed in Table 1. 

In total, 32.04% of participants were in their second year of studies (*N* = 33), 33.01% were in their third year (*N* = 34), and 34.95% were enrolled in their fourth year (*N* = 36), with no statistically significant differences between the group of binge drinkers and non-binge drinkers. Students living with their parents represented 86.41% of the sample. In the case of binge drinkers, 78.72% of the sample lived with their parents, compared to 92.86% of non-binge drinkers, this difference was statistically significant (*p* = 0.037). Regarding the level of father’s studies, these were most often university studies (35.92%) or secondary studies/vocational training (38.83%). In the case of mothers, 33.01% had university studies, and 36.89% had secondary studies/vocational training. No significant differences were found between the groups regarding the level of studies of any of the parents.

The binge drinker participants began drinking at a younger age: 14.80 years [SD 1.81] compared to 15.38 years [SD 1.30] in non-binge drinkers. Furthermore, binge drinkers had been drinking for more years at the time of study: 6.35 years [SD 3.06] compared to 6.06 years [SD 3.51] in the case of non-binge drinkers. Neither of these two differences were statistically significant. The mean amount of alcohol consumed during one day of drinking was 5.97 [SD 4.05] SDUs in binge drinkers compared to 3.81 [SD 2.94] SDUs in non-binge drinkers (*p* = 0.004). 

Smokers represented 14.60% of the total sample. The proportion of BD who smoked was 25.50% compared to 5.40% of non-binge drinkers, with this difference being statistically significant (*p* = 0.004).

Cannabis use was acknowledged by 22.33% of participants. The percentage of binge drinkers who has tried cannabis was 31.90% compared to 14.30% of non-binge drinkers, and this difference was statistically significant (*p* = 0.032).

The level of physical activity was low for 11.65% (*N* = 12) of participants, average for 31.07% (*N* = 32), and high in the case of 57.28% (*N* = 59). No statistically significant differences were found between groups. 

### 3.2. AUDIT Questionnaire.

The responses to the AUDIT questionnaire are displayed in Table 2 and Table 3. We excluded from the analysis the responses provided by those participants who had never consumed alcohol (*n* = 4) or those who considered themselves as non-drinkers (*N* = 7) at the time of study. Thus, a total of 92 questionnaires were included in the analyses. 

Statistically significant differences were found in the three domains of the questionnaire.

Domain 1. Hazardous alcohol use: The most common frequency of drinking in the group of non-binge drinkers was “Monthly or less ” 47.92% followed by “2 to 4 times per month” in 41.67%. Among binge drinkers, the most common frequency of alcohol consumption was “2 to 4 times per month”, 50% followed by “2 to 3 times per week” 29.55% (*p* = 0.003). Up to 25% of binge drinkers consumed six or more alcoholic beverages with a weekly or monthly frequency, compared to 4.17% of non-binge drinkers. The differences in this variable between both groups was statistically significant (*p* = 0.009).

Domain 2. Dependence symptoms: 25% of binge drinkers acknowledged that during the previous year they were unable to perform their normal activities because of drinking one or more times per month, and in 4.55% of participants, this occurred 2 to 4 times per month, compared to percentages of 8.33% and 2.08%, respectively, in the group of non-binge drinkers (*p >* 0.05).

Domain 3. Harmful alcohol use: In the group of binge drinkers, a greater percentage of responses was observed in items 7, 8, 9, and 10, reflecting harmful alcohol consumption, compared to the group of non-drinkers, although this was not statistically significant.

### 3.3. Neuropsychological Tests

No relationship was found between the total scores of the BDI-II between the group of binge drinkers 7.04 [SD 5.66] and the non-binge drinkers: 6.33 [SD 5.81]. Neither was there a relationship found if we categorized this variable into minimal, mild, moderate, and severe. In the scores of the WMS-III Logical Memory I Test, the WAIS-III Digit Span test, and TMT A and B tests, no statistically significant differences were found. The results of the neuropsychological tests are displayed in Table 4.

## 4. Discussion

Our results reveal that over half of students have consumed 60 g and 50 g of alcohol, in the case of men and women, respectively, on a single occasion (over a two-hour period) at least once during the previous 30 days. These results are higher than those reported by Dantzer et al. in the year 2006 [23], on a sample of almost 18,000 university students of 21 countries, between 17 and 30 years of age. In this study, Spain ranks 12th place with a prevalence of consumption of 21% (95%CI 16–25) in the case of men, and 10th place in the case of women, with a rate of 17% (95%CI 12–20). However, our results are similar to those reported by Carbia et al. [13] in 2017 and Salas-Gómez et al., [15] in 2016. Both these Spanish studies were conducted on university students, reporting a prevalence of 50.97% and 47.6% for binge drinking, respectively. Also, in Spain, the EDADES [3] survey, published in 2017, indicated a prevalence of approximately 30% in the case of men and around 16–20% in women aged between 20–29 years.

The differences in reported prevalence rates among these studies may be due to the lack of homogeneity in the concept of binge drinking. Most studies quantify alcohol consumption by the number of drinks instead of SDUs, which may underestimate consumption. Nonetheless, the results reveal that this pattern of alcohol consumption has increased in recent years.

In our sample, the frequency of this pattern of consumption is reduced in students who live with their parents, compared to other forms of cohabitation. This finding coincides with reports by Caamaño-Isorna et al. [24], by Dantzer et al. [23], and Tavolacci et al. [20] who considered this as being a protective factor. This relationship can be explained, on the one hand, due to a greater control on behalf of parents over their children if they live in the same home, as well as the fact that alcohol consumption is considered as a step in the transition to adulthood and emancipated students have already initiated this process.

Several authors have related a high socioeconomic level with this pattern of consumption [21,23,24], however in our sample, no differences were detected between both groups. This may be due to our sample size.

In our sample, the mean age at which respondents drank alcohol for the first time was around 15 years of age. This age was slightly lower in binge drinkers. Other studies involving Spanish university students [15,24] report similar ages of onset. The survey on Alcohol and other Drugs in Spain (EDADES), 1995–2017 [3], raises the mean age to 16.6 years. Hence, although the Spanish legislation prohibits the purchase and consumption of alcohol under the age of 18, the reality is that young people are able to access alcohol with ease.

One-fourth of binge drinking students were smokers, and this was a significant difference compared to non-binge drinkers. This association between BD and alcohol consumption has already been previously shown by other authors [20,26,43]. In this sense, over 30% of binge drinkers in our sample had consumed cannabis at some point. Several authors in different countries report the same association [19,20,26], suggesting that alcohol is often the first drug consumed by teenagers, and, thereafter, in some cases they progress to other drugs.

Despite the fact that physical activity constitutes a healthy activity, this was not associated with a reduced alcohol consumption in our students. Indeed, Tavolacci et al. [20] related physical activity, especially in a group, with greater alcohol consumption, possibly because it is related to the team’s socialization.

It is important to highlight that the participants were nursing students. Thus, it is assumed that they have a greater knowledge and sensitivity towards healthy habits.

This early and intense onset regarding alcohol consumption highlights the special relevance of educational programs on alcohol and drug consumption directed at young people at centers of learning. Nonetheless, among the teenage population, the approach is more complex and must extend beyond education. The perception that teenagers have with regards socially acceptable behavior is closely related to messages conveyed via advertisements and social networks [44,45].

Regarding the responses to the AUDIT questionnaire, the differences in the scores of the three test domains are significant. The ‘hazardous alcohol use’ domain, which evaluates alcohol consumption, displayed statistically significant relationships among some of the items. The domains ‘dependence symptoms’ and ‘harmful alcohol use’, which we could group as being consequences of consumption, did not feature any item in which the difference in scores was significant, however higher scores were obtained in the group of binge drinkers. According to the results of AUDIT, the BD in our sample not only drank more, but also did so more frequently. Their dependence on alcohol limited their normal activities, demonstrating social repercussions of BD. The AUDIT questionnaire found differences among both patterns of consumption.

Lastly, regarding the neuropsychological repercussions, in our students, we were unable to find statistically significant differences. Nonetheless, Carbia et al. [13] found episodic memory deficits in students with a binge drinking pattern of consumption related to the vulnerability of the teenage hippocampus to the neurotoxic effects of alcohol. Furthermore, Nourse et al. [27] not only detected memory losses, but also problems of anxiety and depression. Finally, Salas-Gómez et al. [15] found a lower performance in executive functions, which was related to the age of beginning alcohol consumption, which suggests an accumulative effect of the damage.

Our study presents a series of limitations, which are inherent to cross-sectional designs regarding the interpretation of causational factors. This was a preliminary study based on a sample of only 103 students. Our findings should be confirmed and extended in future large studies. Furthermore, it does not have a control group (non-drinker) to compare with binge drinkers and non-binge drinkers. All participants were enrolled in the Faculty of Nursing, therefore there could have been gender bias.

Furthermore, there is no international consensus for the definition of binge drinking and the measurement of alcohol consumption based on SDUs or number of consumptions. It would be interesting to study the effects of alcohol use in the mid and long term with the aim of evaluating whether the effects are maintained, accumulated, or decreased and whether the effects disappear when maintaining, increasing, or decreasing the pattern of binge drinking alcohol consumption.

## 5. Conclusions

Over half of university students present a binge drinking consumption pattern. Our results show that, despite the sample size, there is an important relationship between binge drinking patterns, place of residence, and tobacco and cannabis use. Nonetheless, to confirm our findings, future studies are needed with larger sample sizes and including a follow-up of participants in the mid and long term.

## Figures and Tables

**Table 1:** Academic, sociodemographic data, alcohol, tobacco, cannabis consumption, and physical activity.

	Total (*N* = 103)		Binge Drinkers			
			No (*N*= 56)		Yes (*N* = 47)	
	*N*	%	*N*	%	*N*	%
Gender						
Female	87	84.47%	49	87.50%	38	80.85%
Male	16	15.53%	7	12.50%	9	19.15%
				*p* > 0.05		
Age	21.21 [SD 2.55]		21.3 [SD 2.93]		21.11 [SD 2.02]	
	*p* > 0.05					
Academic year						
2nd	33	32.04%	21	37.50%	12	25.50%
3rd	34	33.01%	20	35.70%	14	29.80%
4th	36	34.95%	15	26.80%	21	44.70%
	*p* > 0.05					
Mean university access grade	11.11 [SD 0.79]		11.16 [SD 0.83]		11,04 [SD 0.75]	
				*p >* 0.05		
Place of residence						
Family home	89	86.41%	52	92.86%	37	78.72%
Not in the family home	14	13.59%	4	7.14%	10	21.28%
	*p* < 0.05					
Maternal level of studies						
University	34	33.01%	23	41.07%	11	23.40%
Secondary/vocational training	38	36.89%	18	32.14%	20	42.55%
Primary	26	25.24%	13	23.21%	13	27.66%
No studies	5	4.85%	2	3.57%	3	6.38%
	*p >* 0.05					
Paternal level of studies						
University	37	35.92%	18	32.14%	19	40.43%
Secondary /vocational training	40	38.83%	20	35.71%	20	42.55%
Primary	22	21.36%	16	28.57%	6	12.77%
No studies	4	3.88%	2	3.57%	2	4.26%
	*p >* 0.05					
Smoker						
Yes	15	14.60%	3	5.40%	12	25.50%
No	88	85.40%	53	94.60%	35	74.50%
	*p* < 0.05					
Have you ever consumed cannabis?						
Yes	23	22.33%	8	14.30%	15	31.90%
No	80	77.67%	48	85.70%	32	68.10%
	*p* < 0.05					
Do you consider yourself a binge drinker?						
Yes	44	42.72%	14	25.00%	30	63.83%
No	59	57.28%	42	75.00%	17	36.17%
	*p <* 0.001					
Age of onset of alcohol use (*n* = 99) ^1^	15.11 [SD 1.58]		15.38 [SD 1.30]		14.80 [SD 1.81]	
	*p >* 0.05					
Years since the first alcohol consumption (*n* = 99) ^1^	6.19 [SD 3.29]		6.06 [SD 3.51]		6.35 [SD 3.06]	
	*p >* 0.05					
SDUs on a typical day when you were drinking alcohol over the last 12 months (*n* = 92) ^2^	4.84 [SD 3.66]		3.81 [SD 2.94]		5.97 [SD 4.05]	
	*p* < 0.05					
Physical activity (MET-minutes/week)	4324.27 [SD 4240.74]		4280.78 [SD 4133.38]		4376.09 [SD 4409.61]	
	*p >* 0.05					
Physical activity (IPAQ-SF)						
Low	12	11.65%	7	12.50%	5	10.64%
Moderate	32	31.07%	18	32.14%	14	29.79%
Intense	59	57.28%	31	55.36%	28	59.57%
	*p >* 0.05					

^1^ 4 Participants have never consumed alcohol. ^2^ 11 Participants do not currently drink alcohol.

**Table 2:** Alcohol use disorders identification test (AUDIT) questionnaire [38].

Questions	Total (*N* = 92) ^1^		Binge Drinkers			
			No (*N* = 48)		Yes (*N* = 44)	
	*N*	%	*N*	%	*N*	%
1. How often do you have a drink containing alcohol? ^2^						
Never	3	3.26%	2	4.17%	1	2.27%
Monthly or less	31	33.70%	23	47.92%	8	18.18%
2 to 4 times a month	42	45.65%	20	41.67%	22	50.00%
2 to 3 times a week	16	17.39%	3	6.25%	13	29.55%
				*p <* 0.05		
2. How many standard drinks containing alcohol do you have on a typical day when drinking? ^2^						
1 or 2	44	47.83%	26	54.17%	18	40.91%
3 or 4	32	34.78%	15	31.25%	17	38.64%
5 or 6	10	10.87%	5	10.42%	5	11.36%
7 to 9	3	3.26%	1	2.08%	2	4.55%
10 or more	3	3.26%	1	2.08%	2	4.55%
				*p >* 0.05		
3. How often do you have six or more drinks on one occasion? ^2^						
Never	52	56.52%	34	70.83%	18	40.91%
Less than monthly	27	29.35%	12	25.00%	15	34.09%
Monthly	10	10.87%	2	4.17%	8	18.18%
Weekly	3	3.26%	0	-	3	6.82%
				*p <* 0.05		
4. During the past year, how often have you found that you were not able to stop drinking once you had started? ^2^						
Never	77	83.70%	43	89.58%	34	83.70%
Less than monthly	12	13.04%	5	10.42%	7	13.04%
2 to 4 times a month	3	3.26%	0	-	3	3.26%
				*p >* 0.05		
5. During the past year, how often have you failed to do what was normally expected of you because of drinking? ^2^						
Never	74	80.43%	43	89.58%	31	70.45%
Less than monthly	15	16.30%	4	8.33%	11	25.00%
2 to 4 times per month	3	3.26%	1	2.08%	2	4.55%
				*p >* 0.05		
6. During the past year, how often have you needed a drink in the morning to get yourself going after a heavy drinking session the previous evening?^2^						
Never	75	81.52%	43	89.58%	32	72.73%
Monthly or less	11	11.96%	3	6.25%	8	18.18%
2 to 4 times per month	4	4.35%	2	4.17%	2	4.55%
2 to 3 times per week	2	2.17%	0	-	2	4.55%
				*p >* 0.05		
7. During the past year, how often have you had a feeling of guilt or remorse after drinking? ^2^						
Never	53	57.61%	31	64.58%	22	50.00%
Monthly or less	33	35.87%	15	31.25%	18	40.91%
2 to 4 times per month	5	5.43%	1	2.08%	4	9.09%
2 to 3 times per week	1	1.09%	1	2.08%	0	-
				*p >* 0.05		
8. During the past year, have you been unable to remember what happened the night before because you had been drinking? ^2^						
Never	58	63.04%	35	72.92%	23	52.27%
Monthly or less	28	30.43%	12	25.00%	16	36.36%
2 to 4 times per month	6	6.52%	1	2.08%	5	11.36%
				*p >* 0.05		
9. Have you or someone else been injured as a result of you drinking? ^3^						
No	76	82.61%	42	87.50%	34	77.27%
Yes, but not in the past year	12	13.04%	5	10.42%	7	15.91%
Yes, during the past year	4	4.35%	1	2.08%	3	6.82%
				*p >* 0.05		
10. Has a relative, or friend, doctor or other health worker been concerned by your drinking or suggested you cut down? ^3^						
No	85	92.39%	47	97.92%	38	86.36%
Yes, but not in the past year	4	4.35%	0	-	4	9.09%
Yes, during the past year	3	3.26%	1	2.08%	2	4.55%
				*p >* 0.05		

^1^ 11 participants were excluded as they currently do not consume alcohol. ^2^ Response scores 0, 1, 2, 3, 4. ^3^ Response scores 0, 2, 4.

**Table 3:** AUDIT Questionnaire total and AUDIT-C.

	Total (*N* = 92) ^1^		Binge Drinkers			
			No (*N* = 48)		Yes (*N* = 44)	
	Mean	SD	Mean	SD	Mean	SD
AUDIT total	5.13 [3.95]		3.77 [3.01]		6.61 [4.35]	
				*p <* 0.001		
AUDIT-C ^2^ total	3.17 [1.93]		2.50 [1.44]		3.91 [2.12]	
				*p <* 0.001		
AUDIT DOMAINS ^3^						
Domain 1. Hazardous alcohol use	3.17 [1.93]		2.50 [1.44]		3.91 [2.12]	
				*p <* 0.001		
Domain 2. Dependence symptoms	0.70 [1.27]		0.38 [0.98]		1.05 [1.46]	
				*p* < 0.05		
Domain 3. Harmful alcohol use	1.26 [1.53]		0.90 [1.28]		1.66 [1.68]	
				*p* < 0.05		

^1^ 11 participants were excluded as they currently do not consume alcohol. ^2^ The three first questions of AUDIT so called AUDIT-C. ^3^ The domains group questions linked to the same concept: Domain 1 (questions 1–3), Domain 2 (questions 4–6), and Domain 3 (questions 7–10). SD: Standard deviation.

**Table 4:** Neuropsychological tests.

	Total (*N* = 103)		Binge Drinkers			
			No (*N* = 56)		Yes (*N* = 47)	
	Mean	SD	Mean	SD	Mean	SD
BDI-II [42] ^1^	6.66	5.72	6.33	5.81	7.04	5.66
				*p >* 0.05		
BDI-II categories [42]	N	%	N	%	N	%
Minimal (Response score: 0–13)	92	89.32%	50	89.29%	42	89.36%
Mild (Response score: 14–19)	6	5.83%	2	3.57%	4	8.51%
Moderate (Response score: 20–28)	4	3.88%	3	5.36%	1	2.13%
Severe (Response score: 29–63)	1	0.97%	1	1.79%	0	0.00%
				*p >* 0.05		
WMS III Logical memory I [39] ^2^	9.782	2.28	9.82	2.39	9.73	2.17
				*p >* 0.05		
WAIS-III digit span [40] ^3^	20.83	4.81	20.02	4.7	21.79	4.81
				*p >* 0.05		
TMT A [41] ^4^	21.38	7.12	21.54	7.31	21.19	6.97
				*p >* 0.05		
TMT B [41] ^5^	53.95	16.95	55.59	19.61	52	13.04
				*p >* 0.05		

^1^ 21 Items. Response scores: 0–3. Total response score: 63, ^2^ 10 Items. Response correct: 1. Total response score: 10, ^3^ 17 items. Response scores: 0, 1, 2. Total response Score:30, ^4^ Visuo-motor tracking speed. Time in seconds to do the exercise, ^5^ Attention and mental flexibility. Time in seconds to do the exercise.
